# Link-Correlation-Aware Opportunistic Routing in Low-Duty-Cycle Wireless Networks

**DOI:** 10.3390/s21113840

**Published:** 2021-06-01

**Authors:** Xingfa Shen, Lili Liu, Zhenxian Ni, Mingxin Liu, Bei Zhao, Yuling Shang

**Affiliations:** 1 School of Computer Science and Technology, Hangzhou Dianzi University, Hangzhou 310018, China; shenxf@hdu.edu.cn (X.S.); liull@hdu.edu.cn (L.L.); sineyer@hdu.edu.cn (Z.N.); 2 College of Electrical and Information Engineering, Guangdong Ocean University, Zhanjiang 524088, China; liumx@gdou.edu.cn; 3 Shanghai Lilith Technology Corporation, Jiading District, Shanghai 200233, China; billshang@lilith.com

**Keywords:** opportunistic routing, link correlation, low-duty-cycle, expected transmission count

## Abstract

In low-duty-cycle wireless networks with unreliable and correlated links, Opportunistic Routing (OR) is extremely costly because of the unaligned working schedules of nodes within a common candidate forwarder set. In this work, we propose a novel polynomial-time node scheduling scheme considering link correlation for OR in low-duty-cycle wireless networks (LDC-COR), which significantly improves the performance by assigning nodes with low correlation to a common group and scheduling the nodes within this group to wake up simultaneously for forwarding packets in a common cycle. By taking account of both link correlation and link quality, the performance of the expected transmission count (ETX) is improved by adopting the LDC-COR protocol. As a result, the energy consumption of low-duty-cycle OR is significantly reduced. LDC-COR only requires the information of one-hop neighboring nodes which introduces minimal communication overhead. The proposed LDC-COR bridges the gap between the nodes’ limited energy resource and the application lifetime requirements. We evaluate the performance of LDC-COR with extensive simulations and a physical wireless testbed consisting of 20 TelosB nodes. The evaluation results show that both transmission efficiency and energy consumption of low-duty-cycle OR are significantly improved with only a slight increase of end-to-end delay.

## 1. Introduction

Currently, more and more applications (e.g., industrial Internet of Things, structural health monitoring [1] and habitat monitoring [2]) are required to work for a long period of time. As a result, low-duty-cycle wireless networks have become increasingly common due to the increasing gap between rapidly growing lifetime requirements and slow progress in battery capacity [3]. To fill this gap, wireless devices operate in extremely low-duty-cycle in which a node keeps an active state briefly and schedules itself dormant for a long time in a period [4]. As a large number of nodes remain dormant for a long time, the unnecessary energy consumption of idle listening is reduced, and thus the lifespan of network is significantly prolonged.

In low-duty-cycle wireless networks, opportunistic routing (OR) has great potential to improve the network performance by providing each transmission multiple opportunities to make progress without need for more network capacity than traditional routing protocols [5,6]. It exploits the broadcast diversity benefit of wireless networks by choosing a set of nodes as forwarders to transmit the data packets rather than identifying a single forwarder in advance. As soon as at least one node in the forwarder set receives the packet, the sender terminates the transmission. There are significant benefits of reception diversity which can be exploited to improve the network performance.

Existing work on OR in low-duty-cycle wireless networks ignores the impact of reception diversity among the forwarder set, thus the benefits of OR cannot be fully exploited. These studies explicitly or implicitly suppose that wireless links are independent of each other. Recent work, however, has shown clear evidence that wireless links are not independent and packet transmissions from a sender to multiple receivers within a short time interval are correlated, which are mainly caused by cross-network interference and correlated shadowing [7,8,9,10,11]. The lack of consideration of link correlation overestimates the performance efficiency of OR in low-duty-cycle wireless networks.

To fully exploit the diversity benefits of OR in low-duty-cycle wireless networks with the consideration of link correlation, we need to tackle several challenges. Firstly, in the low-duty-cycle mode, the network may have temporary losses of connectivity. A sender spends long period of time (i.e., sleep latency [12]) waiting until the receiver wakes up. The end-to-end delay increases accordingly, resulting in degrading the transmission efficiency. Both sleep delay and unaligned scheduling lead to a significant performance degradation, making OR in low-duty-cycle networks become a new and challenging issue. Secondly, wireless communication links are proven correlated with each other commonly, instead of independent of each other. The combination of low-duty-cycle operation and link correlation makes the problem of OR different from that found in common low-duty-cycle networks or always-awake wireless networks.

In this paper, we propose a link-correlation aware scheduling scheme for OR in low-duty-cycle wireless networks with unreliable and correlated links, called LDC-COR. This scheme consists of two phases: grouping and re-scheduling. In grouping phase, a sender node groups its one-hop neighboring nodes based on the information of link correlation and link quality, making full use of the reception diversity of the candidate forwarder set. In re-scheduling phase, the nodes within the same group are assigned a common awake time slot to align their working schedules so that they can forward the data packets simultaneously. LDC-COR is more efficient in both transmission cost and energy consumption. In summary, our contributions are as follows:

- We comprehensively study the problem of OR with correlated receptions and find that OR prefers links with low correlation. To the best of our knowledge, we are the first to investigate the impact of link correlation on OR in low-duty-cycle wireless networks.
- We propose a novel link-correlation-aware OR protocol, called LDC-COR, for low-duty-cycle wireless networks. LDC-COR leverages a novel candidate forwarder scheduling algorithm to help OR fully exploit the diversity benefit in low-duty-cycle modes.
- We implement and evaluate our design on a real-world testbed with 20 TelosB sensors and by extensive simulations. Both testbed evaluation and simulation results show that our design reduces transmission overhead by 15%∼50% and the energy efficiency is improved by about 30%.

The rest of the paper is organized as follows: Section 2 discusses the related work. Section 3 presents the motivation of this work, followed by the main design of LDC-COR in Section 4. Evaluation results based on simulations and testbed experiments are shown in Section 5 and Section 6, respectively. Section 7 discusses the application scenario and algorithm performance. Finally, Section 8 concludes the paper.

## 2. Related Work

In wireless networks, routing protocol has always played a very important role, which is being responsible for establishing the route from the source node to the destination node and transmitting data reliably. As essential development for wireless networks, opportunistic routing, low-duty-cycle sensor networks and link correlation have been extensively studied in the literature.

In the area of opportunistic routing, Biswas et al. [13] presented ExOR, the first concept of OR, which dynamically chooses a path in a wireless network. Fradj et al. [8] presented a performance analysis of energy consumption referring to opportunistic routing algorithms and disclosed that opportunistic routing algorithm yields a significant improvement of power consumption. Kabaou et al. [14] proposed a comparative study between two opportunistic protocols, which are extremely opportunistic routing protocol and simple opportunistic adaptive routing protocol. Chithaluru et al. [15] presented AREOR, which clustered the network into the optimal number of clusters based on three spatial node density, and nodes spread in a given radius comes in a cluster. Chithaluru et al. [16] presented ARIOR, a recently proposed improved mechanism to determine the Cluster Head(CH) and set of nodes participating in opportunistic routing. The improved routing table was generated based on node ranking for node selection to forward the packets towards the gateway.

In the area of low-duty-cycle sensor networks, Gu et al. [17] presented dynamic switch-based forwarding (DSF) to optimize the transmission efficiency of extremely low-duty-cycle sensor networks and solve the problem caused by unreliable links and sleep latency. Guo et al. [18] proposed Correlated Flooding solving the problem caused by both low-duty-cycle operation and ACK implosion, and achieved satisfactory energy efficiency in flooding design by letting nodes with common parents wake up at the same time. In [19], Duquennoy et al. presented ORPL, an opportunistic routing protocol that supports any-to-any, on-demand traffic and can achieve low-latency data collection in duty-cycled networks. In [20], Ghadimi introduced ORW, a practical opportunistic routing scheme for wireless sensor networks. ORW reduces the delay and energy consumption by utilizing all neighbors as possible next hops, suitable for sensor nodes that frequently enter sleep states to ensure long network lifetime. Chen et al. [21] introduced a novel selective reference mechanism based on spatiotemporal properties of the neighborhood and mobility of nodes. This work reduced discovery delay significantly by proactively referring wake-up schedules among a group of nodes. Wu et al. [22] analyzed the relationship between the duty cycle of the node and the network lifetime and transmission delay and proposed DMADC, a novel scheme named transmission delay minimization based on adjustable duty cycle.

In the area of link correlation, prior work explicitly or implicitly considered the wireless links to be independent. In [23], the correlation between packet losses in wireless links was modeled. However, they incorrectly assumed that the packet receptions are independent. However, recent studies have proved that wireless links are not independent. Srinivasan et al. [7] presented a metric κ to capture the degree of link correlation and showed that link correlation has implications for protocol performance. In [24], Basalamah et al. presented a selection algorithm to help to diversify opportunistic links. Zhao et al. [25] presented CorModel to predict link correlation in low-power wireless networks. Kamari et al. [26] proved that neglecting link correlation will deteriorate the performance of OR protocols. Sharma et al. [27] designed a new contention mechanism exploiting the link correlation, improving the end-to-end throughput and packet reception ratio at each node.

To date, there is much literature [19,20] that has explored OR in low-duty-cycle networks. In addition, wireless communication links are commonly proved to be correlated with each other. However, there are very few studies combining the above three topics at the same time. This paper proposes a link-correlation-aware scheduling scheme for OR in low-duty-cycle wireless networks. While saving energy, our schedule fully exploited the benefits of OR.

## 3. Motivation

### 3.1. Notations

This subsection defines the notations (Table 1).

### 3.2. Impact of Link Correlation on OR

As the core advantage of OR is derived from the reception diversity of data packets among the candidate forwarder set, the key issue to improve the performance of OR is how to correctly select nodes in the candidate forwarding set. Prior work explicitly or implicitly supposes that the wireless links are independent [15,16] and do not take link correlation [23] into account in the candidate forwarder selection process, which just selects the link with the best link quality or randomly. Such strategies do not make full use of the reception diversity of data packets among candidate forwarder set due to their inherent drawbacks.

**Figure 1:**
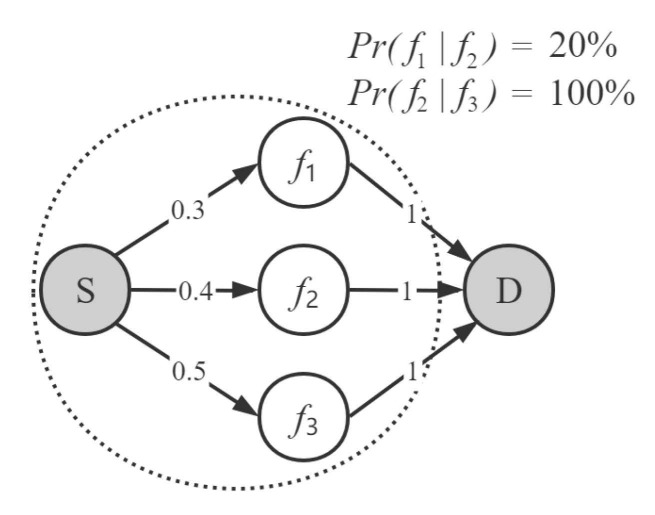
An example of OR in wireless network with link correlation.

We demonstrate how link correlation affects the efficiency of OR. Figure 1 shows a simple two-hop wireless network where *S* and *D* are the source node and the destination node, respectively, while $f_{1}$, $f_{2}$ and $f_{3}$ are three candidate forwarder nodes. In this example, the size of candidate forwarder set is set to 2. The ETX for node $f_{1}$, $f_{2}$ and $f_{3}$ are 11pf1pf1=110.30.3≈3.333, 11pf2pf2=110.40.4=2.5 and 11pf3pf3=110.50.5=2, respectively. Obviously, selecting nodes $f_{2}$ and $f_{3}$ as candidate forwarder set would be the best choice, because they have better link quality compared with those of other combinations.

However, if we considered link correlation, the result of forwarder set selection would be entirely different. With the link correlation model, the ETX of two nodes in the candidate forwarder set received one packet is computed as follows:

(1) $$ETX_{i,j}\left(S\right)=\frac{1}{p\left(f_{i}\right)+p\left(f_{j}\right)-p\left(f_{i},f_{j}\right)}$$

where $p(f_{i},f_{j})$ is the probability that the transmission from sender *S* is received at nodes $f_{i}$ and $f_{j}$ at the same time. From the calculation of conditional probability, we have $p\left(f_{i},f_{j}\right)=p\left(f_{i}|f_{j}\right)*p\left(f_{j}\right)$. Without losing the generality, the number of nodes in the set is more than 2 and the ETX for receiving one packet from sender by at least one node in the forwarder set is given by:

(2) $$ETX\left(S\right)=\frac{1}{\sum_{k=1}^{N}{\left(-1\right)}^{k-1}p\left(f_{1},\ldots ,f_{k}\right)}$$

where $p(f_{1},\ldots ,f_{k})$ is the event transmission from sender *S* is lost at nodes $f_{1},f_{2},\ldots ,f_{k}$ at the same time.

In Figure 1, the ETX for candidate forwarder set $\left\{f_{2},f_{3}\right\}$ is 2.5, while the ETX of set $\left\{f_{1},f_{2}\right\}$ is 1.613. As a result, the candidate forwarder set $\left\{f_{2},f_{3}\right\}$ needs to retransmit more data packets although they have better link qualities. More reception diversity benefits among candidate forwarder set are achieved by selecting the nodes with low correlation as candidate forwarder set [11]. In summary, besides link quality, link correlation is also an important factor for candidate forwarder selection and has a significant impact on the performance of OR.

### 3.3. Impact of Low-Duty-Cycle Network Model

We assume a wireless network consisting of *N* nodes in a given field. Every node is in either an active or a dormant state at time *t*. When a node stays dormant, it just retains a timer so that it wakes itself up according to its working schedule with other functions shut down, so it only receives data packets when it is active. Node’s state switching is based on its own working schedule. Formally, as mentioned in [18], the low-duty-cycle network is defined as a time-dependent graph, representing the potential traffic flows within the network at time *t*.

**Figure 2:**
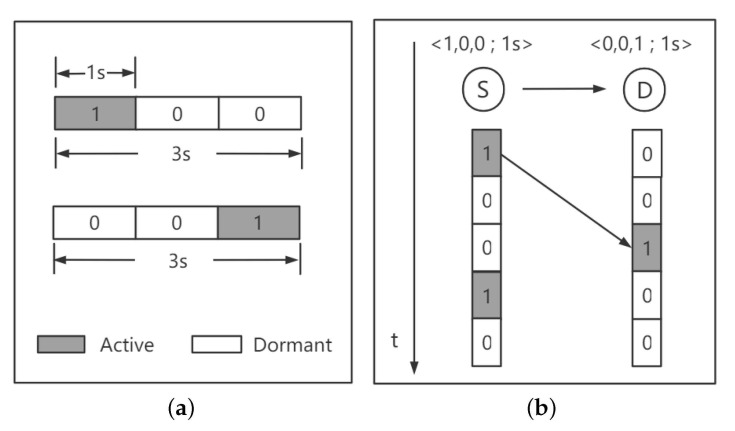
A low-duty-cycle network model. (**a**) Time slots example; (**b**) Data forwarding example in low-duty-cycle mode.

We assume that each node’s working schedule is represented as F=(w,τ), in which *w* is a binary string whose length $\left|w\right|$ indicates the number of time slots of the node in a cycle and each slot’s length is τ. For *w*, the bits ‘1’ and ‘0’ represent active and dormant states, respectively. Normally, the network is periodic, whose period is $T=\left|w\right|*\tau$. Figure 2 shows a simple example of low-duty-cycle network model. Figure 2a shows the example of < 1,0,0; 1 s > and < 0,0,1; 1 s > where T is 3 s and is divided into 3 time slots, each of which is 1s long. As shown in this figure, a node with schedule < 1,0,0; 1 s > is active during the first 1s and dormant during the rest 2 s. Using this model, the delay of a packet can be easily computed and represented by the number of time slots. As shown in Figure 2b, the *w* of node *S* is [1,0,0] and its working schedule has three slots whose lengths are 1 s. Specifically, it is in an active state during the first slot and in dormant states during the next two slots in a period. Assuming sender node *S* would transmit packets to the destination node *D* in the first slot, *S* has to wait for 2 s, respectively, until the receiver node *D* wakes up.

### 3.4. Impact of Unaligned Working Schedules on OR in Low-Duty-Cycle Networks

In low-duty-cycle network model, each node is in either an active or a dormant state at time *t*. When a node stays dormant, it just retains a timer so that it wakes itself up according to its working schedule with other functions shut down, so it only receives data packets when it is active. A node’s state switching is based on its own working schedule.

As previously mentioned, link correlation has great potential to improve the protocol performance of OR. However, due to the low-duty-cycle operation pattern, nodes are randomly assigned by working schedules, and most of them may be unaligned. Therefore, when a sender transmits a packet, the nodes within the same candidate forwarder set cannot be awake at the same time and only a part of them remains in active states for delivering the data packets. We will illustrate how the unaligned working schedules of nodes affect the transmission performance and energy efficiency of OR.

**Figure 3:**
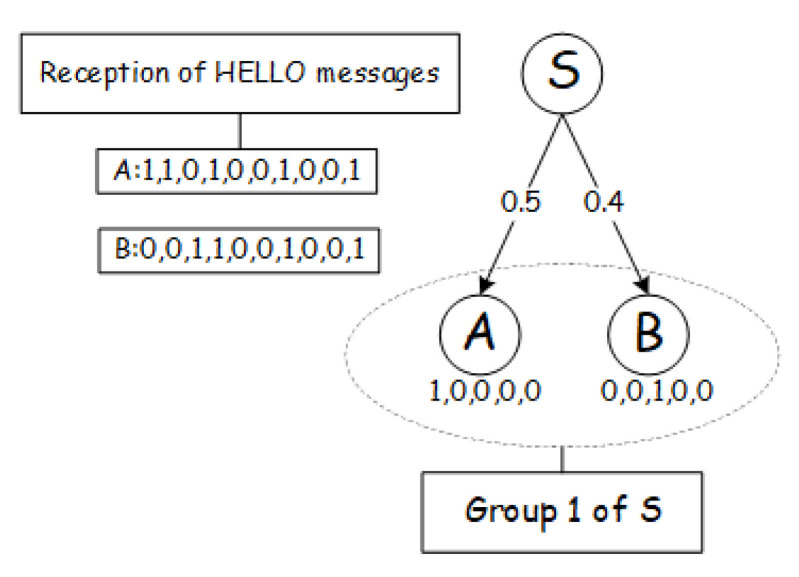
An example of unaligned working schedules.

Figure 3 presents an example of OR in low-duty-cycle networks, where the one-hop neighboring nodes (*A*, *B*) of the sender *S* have already be grouped into one group. The link quality between the sender and receiver is labeled next to the edge connected with them. The box in the upper corner indicates the node’s reception information of the latest 10 HELLO messages, represented by a bitmap, in which ‘1’ indicates successfully receiving the data and ‘0’ indicates missing the data. We use this to calculate the link quality and link correlation in the network, where the link quality and link correlation’s computing methods can be found in Section 4.1. As shown in Figure 3, the number below each node represents its scheduling plan. For example, the work scheduling plan of node *A* in Group 1 is [1,0,0,0,0], which means that its work cycle includes five time slots, and node *A* is scheduled in the first time slot to monitor the channel and perform the forwarding task, and enter dormant state in the remaining time slots. When node *S* sends a data packet to Group 1 in the first time slot, only node *A* is in the awake state and can receive the data packet and forward it, while node *B* is in the dormant state and must wait for two time slots to wake up.

As shown in Figure 3, if the working schedules of nodes in the same group are aligned, we obtain the ETX for Group 1 is 1.667 based on Equation (1) in Section 3.2. However, if the nodes’ working schedules within a same group are unaligned, as marked under them in Figure 3, when node *S* transmits data packets in the first time slot, only node *A* can forward the data packets at this moment because only node *A* is active. Hence, the ETX of the Group 1 is 1/p(A)=1/0.5=2. The totla number of transmissions to complete the whole forwarding task requires to increase by 20%. The reception diversity of candidate forwarder set significantly declines which also brings redundant retransmissions.

## 4. Main Design

**Figure 4:**
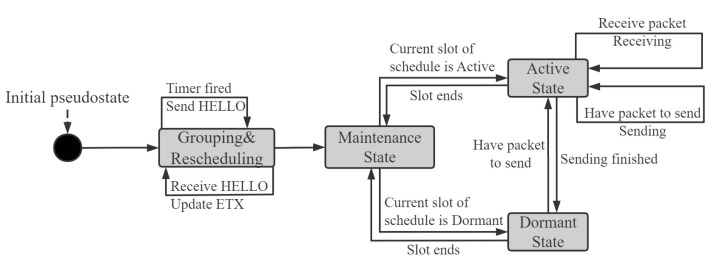
State Machine Diagram of LDC-COR.

This section presents the main design of the LDC-COR, which is a simple finite state machine. As shown in Figure 4, a node running LDC-COR is in one of three states at any time after the initial grouping and rescheduling phase: (i) maintenance state, (ii) active state, and (iii) dormant state. Transitions between the states are triggered by events.

The goal of LDC-COR is to utilize link correlation to maximize the transmission efficiency and minimize the energy consumption of OR in low-duty-cycle wireless networks. In LDC-COR, each node in the network can act as both sender and receiver, which works according to the FSM shown as Figure 4. Each node received the data packet will send an ACK message to the upper-level node. The node will be selected as next-hop whose ACK was received first, continuing to perform data forwarding. The grouping is executed before the sending process. When a node is appointed to forward data packets, the protocol will group all its neighbor nodes, and then use OR to broadcast the data packet to determine the next-hop responsible for forwarding until the data packet is successfully forwarded to the destination node. If several groups select a node as next-hop at the same time, the low-duty-cycle schedule of this node depends on the logical OR(||) operation result of these groups. For example, if Group 1 hopes node A is dormant ($schedule_{1}=0$) and Group 2 hopes node A is active ($schedule_{2}=1$), the schedule of node A will be set to active ($schedule_{1}\left|\right|schedule_{2}=1$).

We divide LDC-COR algorithm into grouping phase and re-scheduling phase. Firstly, the sender collects the information of link quality and link correlation among its one-hop neighboring nodes. In the grouping phase, the sender exploits this link information and depends on the value of *N* and *K* to group its neighboring nodes. In re-scheduling phase, nodes within the same group re-assign working schedules to share the same working schedules to avoid extra costs caused by low-duty-cycle operations and reduce unnecessary energy consumption. The specific process is introduced in the following subsections.

### 4.1. Collecting Link Information

Before grouping the nodes, we need to collect the information of link quality and link correlation in real time. In wireless networks, every node periodically sends out HELLO messages to its one-hop neighboring nodes at an adaptive time interval *t* which is adjusted according to the link’s stability. HELLO messages are used not only for one-hop neighboring nodes discovery but also the link quality and link correlation calculating and updating.

*Calculation of link quality:* The calculation of link quality is conducted simply by dividing the total number of ‘1 s’ by the length of the bitmap. For example, the link quality represented by the bitmap [1,0,1,0,1] is 3/5 = 0.6.

*Calculation of link correlation:* The calculation of link correlation adopts the conditional probability to indicate the correlation, as shown in a special link-correlation-aware design (please refer to paper [11] for the detailed certification process). The degree of correlation is calculated by dividing the number of ‘1 s’ at the same bits in both $f_{i}$ and $f_{j}$’s reception bitmaps by the length of the bitmap, which is the following equation:

(3) $$P_{r}\left(f_{k}\right)=\frac{1}{L}\sum_{j=1}^{L}B_{f_{1}}\left(j\right)\&...\&B_{f_{k}}\left(j\right)$$

where *L* is the length of bitmap, $B_{f_{i}}\left(j\right)$ represents the receiving situation of node $f_{i}$ for the *j* data packet. If the data packet is successfully received, $B_{f_{i}}\left(j\right)=1$, otherwise $B_{f_{i}}\left(j\right)=0$. For example, $f_{1}$ and $f_{2}$’s bitmaps are [1,0,1,0,1] and [0,1,1,0,1], respectively. The correlation is calculated as: $\frac{1}{5}\left(1\&0+0\&1+1\&1+0\&0+1\&1\right)=40\%$.

### 4.2. Grouping Phase

Every node divides its one-hop neighboring nodes into groups based on the information of both link correlation and link quality. Nodes within the same group are low correlated so that the reception diversity of the group is exploited. As a result, the probability that nodes within the same group failed to receive the packet is quite low when the group has multiple nodes with low correlation.

After collecting the information of link quality and link correlation, each sender divides its one-hop neighboring nodes into multiple groups. The number of nodes of each group is determined by the current data successful acceptance rate (DSAR) of each group, and we set a threshold *r* for this value. The calculation of DSAR is actually 1/ETX. The calculation method of ETX is detailed in Section 3.2. We should avoid grouping the nodes with high correlation into one set because the strength of OR mainly comes from the reception diversity of the candidate forwarder set.

First, we sort one-hop neighbor nodes of the candidates according to link quality. From Equation (3), it is a high probability for nodes with high link qualities to have high correlations with each other. If *N* is greater than or equal to *K*, we choose the top *K* nodes with the highest link quality as the initial nodes of each group. The remaining N−K nodes are assigned to each group as candidate nodes. Then we calculate the minimum ETX based on link correlation for grouping. If *N* is less than *K*, we divide it into *N* groups.

**Figure 5:**
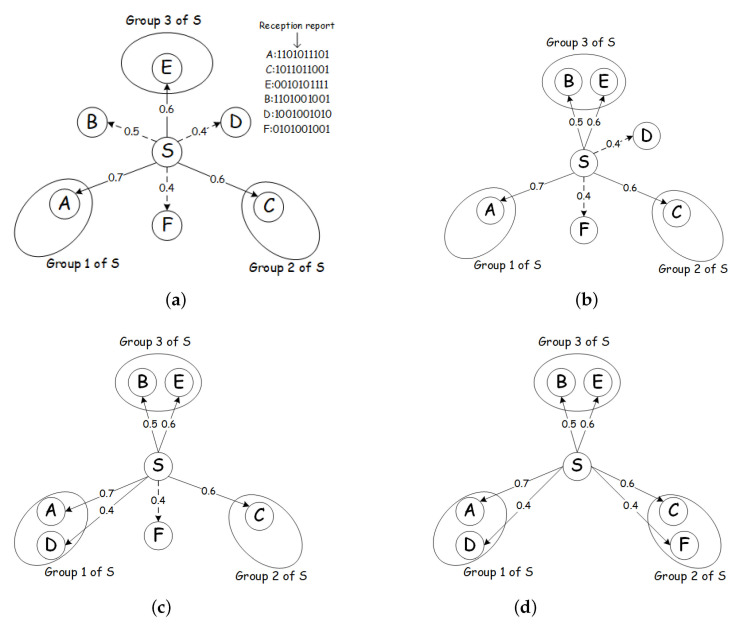
An example of grouping phase. (**a**) Allocate the first node in each group; (**b**) Assign Node *B* to Group 3; (**c**) Assign Node *D* to Group 1; (**d**) Assign Node *F* to Group 2.

Figure 5 shows an example of grouping, where sender *S* divides its six one-hop neighboring nodes into 3 groups with the threshold *r* of DSAR is set to 0.8. As shown in Figure 5a, we first sort the link qualities of one-hop neighboring nodes. Nodes *A*, *C*, and *E* are allocated as the initial nodes of each group, respectively, since they have the highest link qualities among six neighboring nodes. The remaining three nodes are sorted by *B*, *D*, *F* according to their link quality. As for node *B*, adding to three groups, respectively, for comparison, the ETX and DSAR of Group {A,B} are 1.429 and 0.7. Similarly, the ETX and DSAR of Group {C,B} are 1.429 and 0.7, the ETX and DSAR of Group {E,B} are 1.111 and 0.9. Obviously, we should assign node *B* to Group 3, as shown in Figure 5b. As the DSAR of Group 3 reaches the threshold *r*, no more nodes will be added into Group 3. Unless each group’s DSAR reaches the threshold, then re-compared, and the node to be grouped is added to the group with the smallest ETX. As for node *D*, similar to node *B*, the ETX and DSAR of Group {A,D} are 1.25 and 0.8, the ETX and DSAR of Group {C,D} are 1.429 and 0.7, respectively. So node *D* is assigned into Group 1 as shown in Figure 5c, and the DSAR of Group 1 reaches the threshold. Finally, node *F* is allocated into Group 2. The final results of the grouping are shown in Figure 5d.

### 4.3. Re-Scheduling Phase

**Figure 6:**
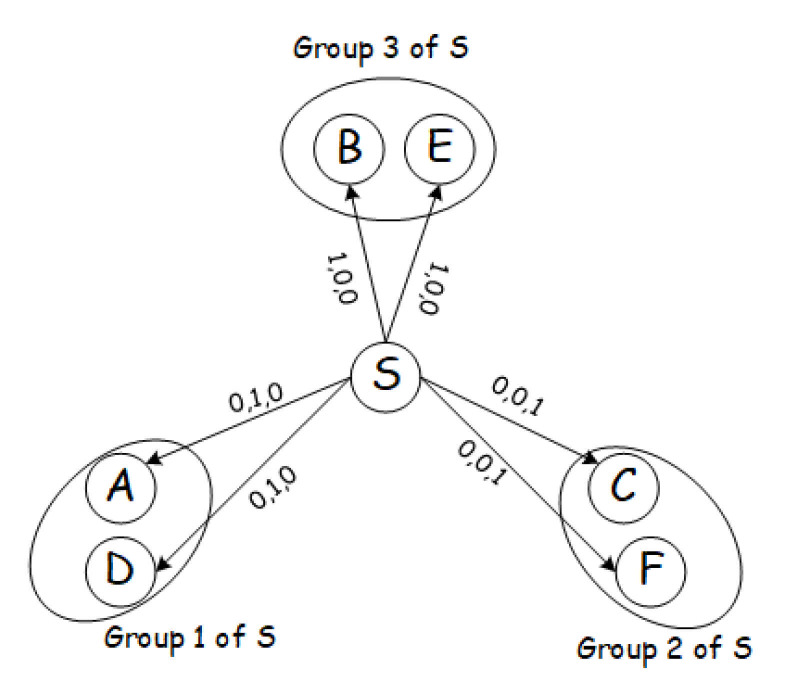
An example of re-scheduling phase.

The working schedules of the nodes within the same group must be adjusted so that they have common active time slots and wake up for receiving and forwarding the packet of OR simultaneously as shown in Figure 6. The specific re-scheduling scheme adopts an aligned wake-up mechanism for the nodes in the same candidate forwarding group, and an unaligned wake-up mechanism for nodes in multiple candidate forwarding groups. In this way, nodes with low correlation wake up at the same time to forward data packet. Only when no nodes in the same group receive the data packet, the sender *S* resends the packet. Nodes receiving a packet decide whether to forward it or not and send a link-layer acknowledgment only if they choose to act as the next hop.

From the example above, both the transmission efficiency and energy consumption are significantly improved by considering link correlation. Specifically, the main procedure of the LDC-COR algorithm is described by the pseudo-code shown in Algorithm 1.

**Algorithm 1** Pseudo-Code of LDC-COR
**Initially,** U(s)←sortN(s); min←∞; index←0; r←0.8; flag←true1:**for**i=0 to *K* **do**2:   group_i(i)←U(i)3:   center(i)←U(i)4:   DSAR(i)←LinkQuality(U(i))5:
**end for**
6:
candidate(s)←N(s)−center(s)
7:**for**i=0 to $\left|candidate(s)\right|$ **do**8:   **for** J=0 to $\left|K\right|$ **do**9:      **if** DSAR(j)<=r **then**10:         flag←false11:         candidate(i)addintogroup_j12:         calculateETXofgroup_jaccordingtoEquation213:         **if** ETX<min **then**14:            min←ETX15:            index←j16:         **end if**17:         group_jremovecandidate(i)18:      **end if**19:   **end for**20:   **if** j==(|K|−1) && flag **then**21:      **for** k=0 to $\left|K\right|$ **do**22:         candidate(i)addintogroup_k23:         calculateETXofgroup_kaccordingtoEquation224:         **if** ETX<min **then**25:            min←ETX26:            index←k27:         **end if**28:         group_kremovecandidate(i)29:      **end for**30:   **end if**31:   candidate(i)addintogroup_index32:   DSAR(index)←1/min33:
**end for**
34:Re-scheduling the working schedules of all groups.


## 5. Simulation

In this section, we evaluate the performance of LDC-COR by using extensive simulations, and compare its performance with other three solutions described as follows:

- **LDC-OR**: LDC-OR does not take account of link correlation while nodes within the same group have aligned working schedules.
- **LDC-ACOR**: LDC-ACOR considers link correlation in the grouping phase while nodes within the same group have unaligned working schedules.
- **LDC-AOR**: LDC-AOR does not consider link correlation while nodes within the same group have unaligned working schedules.

Three metrics are used to evaluate these protocols, which are listed as follows:

- **Transmission Overhead**: The ETX from the sender to the receiver.
- **Time Delay**: The time delay for the receiver receiving a packet successfully from the sender.
- **Energy Consumption**: The energy cost of the network includes sending or receiving packets and waiting for the receiver to wake up.

### 5.1. Simulation Setup

The simulations are conducted on OMNET-4.6, in which we randomly deployed 150 nodes in a given 1000 m × 1000 m field. The communication range of each node is set to 220 m. The first 40s of the simulations are conducted for the network initialization, and the node in the initialization phase only broadcasts HELLO data packets for the maintenance of neighbor information. The candidate forwarding *K* is 3, link quality is 0.6 by default. The threshold *r* of DSAR is set to 0.8. In fact, it does not make much sense for DSAR of each group to exceed 0.8, because retransmission events can only be reduced but cannot be avoided. In addition, sending a packet costs 0.144 W energy, receiving a packet from its sender costs 0.1296 W energy, and the energy cost of idle waiting is 0.00002 W. In the simulation, using different messages to control the progress of the experiment. For example, the “initial self-message” initializes the network. After the node is initialized, it sends “initial self-message” to itself. When the node receives its own “initial self-message”, it starts to send HELLO packets to all neighboring nodes. When a node successfully receives 10 HELLO data packets, it sends “neighInformation self-message” to itself to count link information. After all nodes have received 10 HELLO data packets, the node that sent the data packets sends “prepareOK self-message” to itself to begin routing simulation. We set flags and send multiple self-messages from the starting node to ensure that the comparison is under the same network model. To reduce the influence of randomness in experiments, each result is obtained averaged over 30 runs. The simulation source code and experiment results can be found in the Github [28].

### 5.2. Impact of Link Quality

**Figure 7:**
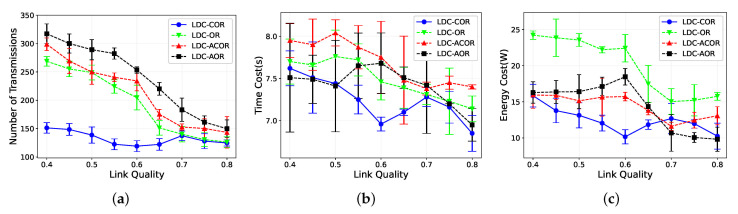
Impact of different link qualities. (**a**) Transmission Overhead; (**b**) Time Delay; (**c**) Energy Consumption.

In Figure 7, we analyze the protocol performance under different link qualities. The link quality varies from 0.4 to 0.8. We add error bars for these four protocols. As shown in Figure 7, the error bar is the variance of the experiment results. Figure 7a shows that the ETX decreases as the link quality increases for all the schemes. Data packets sent by LDC-COR are significantly less than other schemes when the link quality is low, while the advantage of LDC-COR becomes not obvious when the link quality becomes larger. This is because the positive correlation of nodes within the same group would increase and there is little benefit of reception diversity to be exploited when the average link quality increases. Therefore, the performance of LDC-COR is similar to other schemes. Due to the fact that wireless transmission is notoriously unreliable and the link quality is generally low, LDC-COR is more suitable for low-duty-cycle wireless networks compared with other schemes.

Figure 7b shows that the time delays decrease for all schemes as the link quality increases. This stems from the fact that the higher the link quality is, the fewer retransmissions all schemes need. Figure 7c shows that the energy consumption for all schemes decreases as the link quality increases. The reason is that the higher link quality is, the less the energy consumption on redundant retransmissions is.

### 5.3. Impact of Network Size

**Figure 8:**
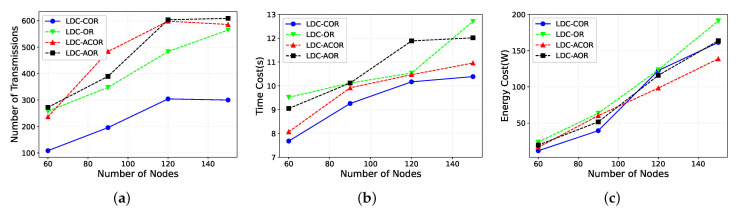
Impact of different network sizes. (**a**) Transmission Overhead; (**b**) Time Delay; (**c**) Energy Consumption.

First, we analyze the network performance for all schemes by varying the number of nodes from 60 to 150, given the condition the network side length is constant. Figure 8a shows that the result of LDC-COR saved about 30% data packets than LDC-OR, LDC-AOR and LDC-ACOR. As the network size increases, the sender has more one-hop neighboring nodes and the group would have more candidate nodes. Thus, more transmissions are saved compared with those schemes that do not take link correlation into account in the grouping process because the reception diversity of the group increases. In addition, unaligned working schedules are the other main reason leading to the decrease of reception diversity.

Figure 8b shows that time delay increases as the network size becomes larger. LDC-COR and LDC-ACOR have similar curves because they both consider the link correlation, but the nodes within the same group under LDC-ACOR scheme may not wake up at the same time, affecting the efficiency of transmission. In summary, LDC-COR is slightly better than other schemes. The reason is that when the number of nodes increases, the hops may increase and the number of nodes within the group would increase. So more reception diversity benefits of candidate forwarder set are exploited. In addition, LDC-COR lets nodes within the same group forward packets simultaneously, which reduces the time overhead.

Figure 8c shows the energy consumption performance of the four schemes for networks with different network sizes. In the beginning, LDC-COR shows more efficient performance. However, with the increase of the nodes, the energy consumption is slightly higher than two unaligned scheduling schemes. The reason is that when the size of the network increase, more nodes miss the timing of forwarding packets, so unaligned scheduling schemes save more energy than aligned scheduling scheme. However, these unaligned scheduling schemes have significant influences on the reception diversity of candidate forwarder set, thus affecting the transmission efficiency where the sender has to retransmit more packets. Therefore, there is a trade-off between unaligned working scheduling and reception diversity of the group. Only considering unaligned scheduling to reduce energy consumption without taking reception diversity into account may result in a higher transmission cost.

### 5.4. Impact of Network Density

**Figure 9:**
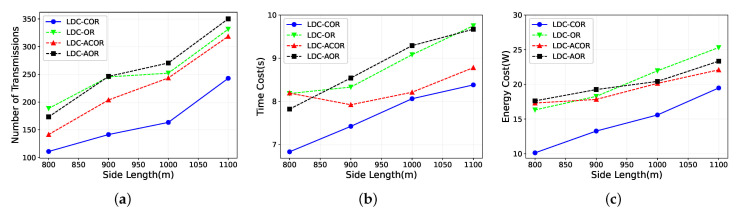
Impact of different network densities. (**a**) Transmission Overhead; (**b**) Time Delay; (**c**) Energy Consumption.

Figure 9 shows the transmission and energy performance for all schemes by varying the side length from 800 m to 1100 m in which the number of nodes is a constant. In Figure 9a, the number of data packets sent increases with the side length of the network increases. As the side length of the network increases, the network density decreases, and sender has fewer one-hop neighboring nodes to deliver the data packets simultaneously. However, LDC-COR still saved about 25∼30% of data packets compared with other schemes.

Figure 9b shows the time delay increases when the density of network decreases since when the network becomes sparser, there are fewer nodes within the same group. As a result, the sender has to spend more time transmitting more packets. LDC-OR and LDC-AOR perform worst here as both of them do not take link correlation into account in the grouping phase but adopt an inefficient random candidate selection or just consider link quality. Figure 9c shows the energy consumption of all the designs increases as the network gets sparser. LDC-COR costs more energy than LDC-ACOR, because as the network becomes sparser, the reception diversity of the group has not been fully exploited yet. LDC-COR also performs better than LDC-AOR and LDC-OR which do not consider link correlation in protocols design.

### 5.5. Impact of Different *K*

**Figure 10:**
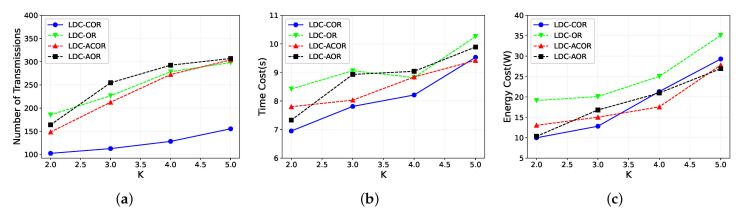
Impact of different *K* values. (**a**) Transmission Overhead; (**b**) Time Delay; (**c**) Energy Consumption.

As shown in Figure 10a, we change *K*, the number of groups, from two to five. The number of packets sent for four schemes increases as *K* increases, while the total number of transmissions only increases slightly in LDC-COR. This is because the candidate selection is becoming inefficient when *K* increases, given the condition the number of nodes is constant. So each group just contains fewer nodes. However, LDC-COR considers both the link correlation and unaligned scheduling, therefore it just has a slight increase compared with other schemes. Similarly, the time cost and energy consumption increase when *K* increases since a lot of retransmissions require more time and energy as shown in Figure 10b,c. With a comprehensive analysis of Figure 8, Figure 9 and Figure 10, LDC-COR is the best choice for low-duty-cycle wireless network compared with the schemes without considering link correlation and unaligned scheduling.

## 6. Testbed Experiments

**Figure 11:**
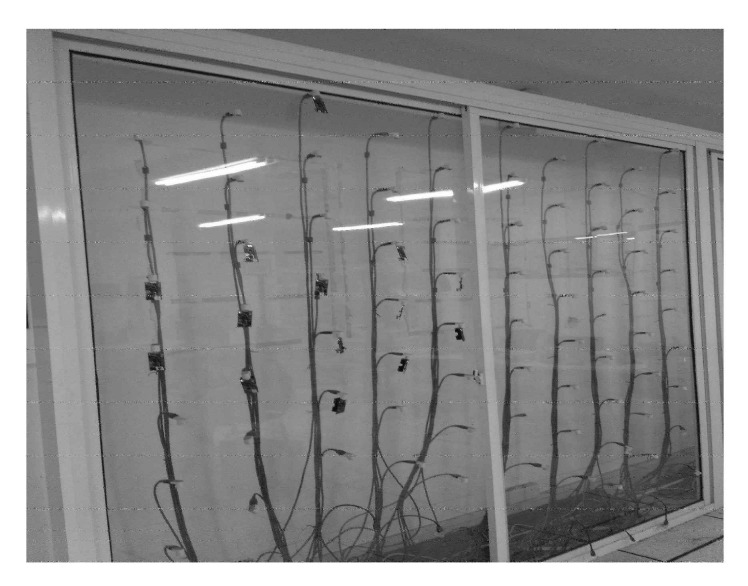
Indoor WSNs testbed.

We implement the schemes on TinyOS 2.1.0 platform, where 20 TelosB sensors are randomly deployed on in-door testbed, as shown in Figure 11. The TelosB node used in the experiment has the characteristics of small size, light weight, low cost and low energy consumption. The TelosB node is shaped like a U disk, using TinyOS as its operating system, and ZigBee and IEEE 802.15.4 as its communication and MAC layer protocols. We implemented and programmed the schemes into the node. The main TinyOS components used in our implementation are MainC, LedsC, ActiveMessageC, CollectionC, AMSenderC, AMReceiverC, TimerMilliC, CC2420PacketC.

### 6.1. Indoor Experiment

In the experiment, we implement the schemes on a 2 m × 4 m indoor testbed, where 20 TelosB sensors are randomly deployed. TelosB node uses an internal 2.4 GHz Planar Inverted Folded Antenna (PIFA) built into the printed circuit board and tuned to match the radio circuitry. The C2420 datasheet specifies that the transmit power can be programmed between −25 to 0 dBm in eight steps. In our experiments, the transmission power level of the sensor nodes is set to 2 to ensure that multi-hop network topology can be formed, whose TX power is less than −25 dbm [29]. Each node rebroadcasts its first-receiving packets exactly once in one time slot. We still use the above three metrics to compare the performance of these four OR schedules. To reduce the influence of randomness in experiments, each result is obtained averaged over 30 runs.

**Figure 12:**
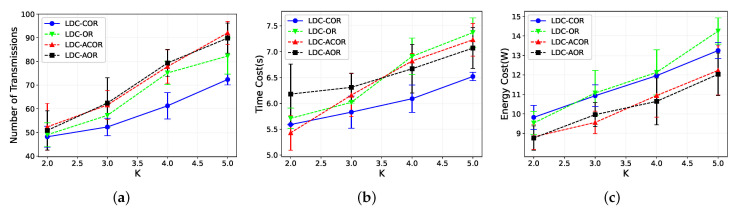
Impact of different *K* values. (**a**) Transmission Overhead; (**b**) Time Delay; (**c**) Energy Consumption.

Figure 12 shows the performance for all schemes by varying *K* from 2 to 5. We add error bars for these four protocols. As shown in Figure 12, the error bar is the variance of the experiment results. Figure 12a shows that when *K* increases, the number of packets sent by all schemes increases, and LDC-COR saves about 20% of transmissions compared with other schemes because, in this small network, each group has fewer nodes to forward the packets as *K* increases. For the time cost shown in Figure 12b, a similar trend is shown since when *K* increases, more time is devoted to retransmission. For energy consumption in Figure 12c, LDC-COR is 13% higher than unaligned designs because, in unaligned schemes, parts of nodes are still sleeping in the transmission process to save some energy consumption. However, it may cause retransmissions, so there exists a trade-off.

### 6.2. Island-Node Observation

While conducting real-world experiment, we observe an island-node phenomenon and propose a practical method to reduce unnecessary transmissions and additional energy consumption induced by them. When there is a low probability of receiving data packets between a node and its neighbor nodes in the wireless network, the receiving node keeps generating useless rebroadcasts, which seriously affects the experimental results. We consider a node exhibiting this observation as an island-node.

In the real-world implementation, to address the island-node problem, we set a limit on the number of retransmissions for each node avoiding several island-nodes lead to high number of rebroadcasts and long end-to-end delay of the whole network. The experimental results show that this solution with a retransmission-limit works well, which significantly reduces the number of useless transmissions by island-nodes while high reliability still can be achieved.

## 7. Discussion

### 7.1. Application Scenario

When there are few nodes, each node has relatively few neighboring nodes, so the advantages of the candidate forwarding set designated by the LDC-COR are difficult to show. In this situation, the performance of the four OR schedules is basically the same. However, when the network is large, the LDC-COR algorithm fully demonstrates its advantages.

### 7.2. Computing Costs

The calculation goal in our algorithm is to make each group perfect so that the diversity characteristics of the group are maximized. It is precisely the group decision that causes the calculation cost. Therefore, we found the theoretical optimum and proved the optimal approximation of these two results through many experiments.

#### 7.2.1. Theoretically Optimal

The optimal theory is full permutation algorithm (FPA).

(4) $$FPA_{m,n}=n(n-1)(n-2)...(n-m+1))=n!(n-m)!$$

As stated in Equation (4), if we want to choose one element from *n* elements, there are *n* ways; if we want to choose another one from the remaining n−1 elements, there are n−1 ways; so that two elements are randomly selected from *n* elements with a total of n∗(n−1) ways. Similar to our grouping example, *n* elements are divided into *K* groups. The number of neighbor nodes in the first group may be any one of 1 to n−K. At the same time, after determining the number *x* of elements in the group, there will be $FPA_{x,n}$ combination methods for selecting *x* nodes to be assigned to the first group from *n* elements, which will cost a huge exponential time complexity. There are also the same ways to determine the second group node, the latter case depends on the previous grouping result, and there is also such an exponential complexity. The algorithm complexity of FPA is O(n!).

**Figure 13:**
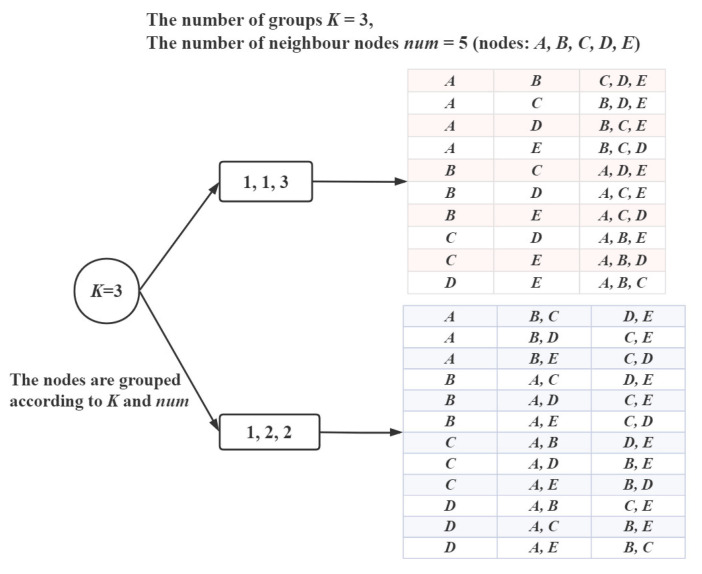
Grouping example by FPA.

We appropriately optimized the existing FPA algorithm, in which first we allocate the number of neighbor nodes in each group. For example, as Figure 13 shows, if there are five neighbor nodes that need to be divided into three groups, firstly we divide the number ‘5’ into three groups, ‘113’ and ‘122’, then group the nodes according to it. Finally, nodes are divided into *K* groups. We calculate the sum of ETX for each grouping situation, then we find the smallest grouping scheme. Although we optimize the FPA, there still exists repeated calculations. The time delay problem is very serious. Therefore, we regard it as the theoretical optimal, and seek other solutions to approximate this optimal result.

#### 7.2.2. Greedy Strategy in LDC-COR

**Figure 14:**
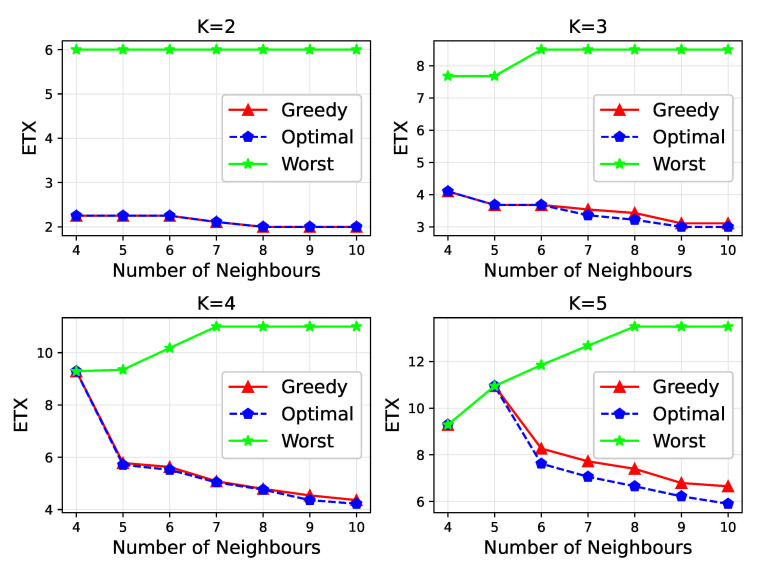
Grouping example of greedy algorithm and theoretically optimal.

The greedy strategy in LDC-COR is our proposed grouping method based on link correlation, whose detail we have introduced in Section 4.2, and its process is shown in Figure 5. The greedy strategy is dividing groups by the nodes’ link correlation and the DSAR of each group. Compared with the exponential time efficiency solution FPA, the greedy strategy as a polynomial algorithm greatly improves the efficiency. Not only the actual performance is good, but the programming of the sensor node is also easy. Figure 14 shows grouping examples of greedy algorithm and the FPA.

Figure 14 shows the performance for two grouping solutions for 10 nodes by varying *K* from 2 to 5. ‘Worst’ refers to the maximum ETX obtained from the FPA, and ‘optimal’ refers to the minmum ETX obtained from the FPA. As shown in Figure 14, the ETX obtained by the greedy algorithm in LDC-COR is relatively close to the theoretical optimal, and has reached the theoretical optimal many times.

## 8. Conclusions

In this paper, we propose LDC-COR, a novel link-correlation-based node scheduling scheme for OR in low-duty-cycle wireless networks that solves the problem caused by low-duty-cycle operations. One important characteristic distinguishes our work from previous work is that, in addition to link quality, we also explore link correlation feature to increase the reception diversity of candidate forwarder set, and let nodes within the same group wake up simultaneously to forward the data packets. LDC-COR is evaluated by extensive simulations and real-world experiments, whose results show that LDC-COR significantly improves both transmission and energy efficiency 15∼50% and about 30% compared with designs without taking account of link correlation, respectively. In future work, we hope the number of groups *K* and the threshold of DSAR *r* can be determined dynamically.

## Figures and Tables

**Table 1:** Notations.

Variables	Definitions
*S*	The source node
$f_{i}$	The candidate forwarder node
$p\left(f_{i}\right)$	The probability that node $f_{i}$ successfully receives the
	packet sent by source node *S*, $p\left(f_{i}\right)\in \left(0,1\right]$
	The probability that node $f_{i}$ successfully receives the
$P_{r}\left(f_{i}|f_{j}\right)$	data packet given the condition that the packet is
	already received by node $f_{j}$
ETX	The expected transmission count (ETX)
*N*	The number of nodes
*F*	Working schedule of each node
	A binary string whose length $\left|w\right|$ indicates the number
*w*	of time slots in a cycle of the node
τ	Length of each slot
*T*	Length of network period
*K*	The number of groups
*r*	The threshold of data successful acceptance rate

## Data Availability

The simulation source code and experiment results can be found in the Github.
